# Allometric equations for estimating belowground biomass of *Androstachys johnsonii* Prain

**DOI:** 10.1186/s13021-015-0027-4

**Published:** 2015-07-25

**Authors:** Tarquinio Mateus Magalhães

**Affiliations:** Departamento de Engenharia Florestal, Universidade Eduardo Mondlane, Campus Universitário, Edifício no.1, 257, Maputo, Mozambique

**Keywords:** Mecrusse, Anchorage, Additivity, Belowground biomass allocation patterns, Root components

## Abstract

**Background:**

The belowground component of the trees is still poorly known because it needs labour- and time-intensive in situ measurements. However, belowground biomass (BGB) constitutes a significant share of the total forest biomass. I analysed the BGB allocation patterns, fitted models for estimating root components and root system biomasses, and called attention for its possible use in predicting anchoring functions of the different root components.

**Results:**

More than half and almost one third of BGB is allocated to the lateral roots and to the root collar, respectively. More than 80% of the BGB is found at a depth range of 9.6–61.2 cm. As the tree size increased, the proportion of BGB allocated to taproots decreased and that allocated to lateral roots increased. All independent models performed almost equally, with the predictors explaining, on average, 98% of the variation in the BGB.

**Conclusions:**

It was hypothesised that BGB allocation patterns are a response of the anchoring functions of the tap and lateral roots and therefore, root component biomass models can be used as a methodology to predict anchoring functions of the different root components. Based on the fact that all models performed almost equally, the models using either diameter at breast height (DBH) exclusively as a predictor should be preferred, as tree height is difficult to measure. Models using the root collar diameter (RCD) only should be preferred when the tree is found cut down, as sometimes the RCD is affected by root buttress. Given the large sample size, the validation results, and the coverage of a wide geographical, soil and climatic range, the models fitted can be applied in all *A. johnsonii* stands in Mozambique.

## Background

*Androstachys johnsonii* Prain (*A. johnsonii*) stands, known as mecrusse, are very important woodlands. Almost entirely restricted to Mozambique [1], it has an important socioeconomic value to local communities, that sell and use stakes and poles of *A. johnsonii* in the construction of homes, shelters, and furniture; and it is the main source of income in the Funhalouro and Mabote districts [2, 3]. On the global scale, mecrusse forests form part of the woodland belt that stretches over large portions of southern Africa and are reported to be a tipping point in regional ecological and socioeconomic development [4], hence, their importance in the mitigation of greenhouse gas emissions.

Forest biomass is a key variable employed when making estimates of carbon pools in forests, and for studying other biochemical cycles [5]. In the past, only the aboveground portion of trees was the desired products from forests [6]. However, with the increased significance of biomass estimation since the Kyoto Protocol was adopted in 1997 [7], and thus, the enhanced awareness of the sequestration functions of trees, climate change issues, have made belowground biomass (BGB) more relevant.

Despite the recent advances in examining root distribution and biomass with ground-penetrating radar [8–12], the belowground component of trees is still poorly known because, traditionally, it requires labour- and time-intensive in situ measurements [13]. Yet, BGB constitutes a major share of total forest biomass. Cairns et al. [14] and Litton et al. [15] have maintained that BGB may represent up to 40% of the total biomass. In Mozambique, Magalhães and Seifert [16, 17] found that approximately 20% of the forest biomass of mecrusse woodlands was allocated to the root system, and so highlighting the need to study this carbon pool.

Besides the share of BGB in whole tree forest biomass, BGB happens to be a unique carbon pool because after exploitation, the root system, along with the stump, are left in the forest and, in some tree species, are then allowed to sprout, continuing the carbon sequestration process or decompose, releasing CO_2_ and nutrients. Therefore, BGB can be used to estimate the carbon that will be transferred to the soil and the nutrients that will be reclaimed by the site.

BGB is often estimated indirectly, using root-to-shoot ratios (R/S) [17–21], root system biomass expansion factors (BEFs) [17], and by using regression equations of BGB versus aboveground biomass (AGB) [18, 22, 23] or versus easily measured variables (diameter at breast height (DBH) and tree height (TH)) [23, 24]. However, whatever the method utilised to estimate BGB (R/S, BEFs, equations), it is necessary that the root system is directly measured to develop those methods.

Based on the fact that measuring BGB is difficult and time-consuming, the root system is often partially removed from the soil [25–30], depths of excavation are predefined [29–31], and fine roots are excluded [19, 32, 33]. However, the depths of excavation and the definition of fine roots are not standardised [18, 34], but the depth selected in a given study is assumed to capture a large proportion of the roots [18]. Yet, according to Mokany et al. [20], sampling to what may be deemed an insufficient soil depth to capture the majority of the roots, while not sampling the root collar or fine roots, as well as sampling with inadequate replication, are a few of the methodological pitfalls associated with sampling root biomass, and can lead to underestimation.

In other cases, a root sampling procedure is applied where only a fraction of roots from each root system are fully excavated, and then the information from the excavated roots is employed to estimate biomass for the roots not excavated [6, 23, 35]. The disadvantage of relying on sampling procedures is that the observed biomass value for each individual root system is less accurately determined compared to excavating in full [6].

Very few allometric biomass models exist for Mozambican forests; exceptions include Magalhães and Seifert [16, 17], Ryan et al. [33], Mate et al. [36], and Sitoe et al. [37]. As is best present known, the only studies that have included BGB are those by Magalhães and Seifert [16, 17], Ryan et al. [33], and Magalhães and Seifert [38]. However, the study by Ryan et al. [33] was based on only several sample trees (23) within a limited geographical range (27 ha) and the root system was not completely excavated (fine roots were not included). Although Magalhães and Seifert [16, 17, 38] considered relatively large sample trees and an expanded geographical area (93 trees harvested in 5 districts), besides including the entire root system, their allometric models were limited by not considering the different root components (e.g. taproot, root crown, lateral roots) and, therefore, the BGB allocation patterns were not analysed. Root component biomass models and BGB allocation patterns analyses are scarce worldwide, Litton et al. [15] being the only reference available in the literature.

Studying BGB allocation patterns is very important for understanding root anchorage as both root anchorage and BGB allocation patterns depend on root architecture, branching patterns, and size and depth of the roots [39–42]. In turn, those factors affecting tree anchorage and BGB allocation patterns depend on tree species and soil types [43] and resources [44–48]. The anchoring capacity of a tree is a critical factor for survival regarding external abiotic stresses [49].

It has been suggested by Herrel et al. [49] that for a fixed amount of biomass, a network of several small roots is more resistant to tension than a few large structural roots. This means that a root system with a larger taproot and several smaller lateral roots, as in the case of *A. johnsonii* trees [50], is more resistant to tension and therefore may have a better anchoring capacity than otherwise.

Parametric studies have shown that the number of lateral ramifications and their diameter were both major components affecting the resistance to pull-out for a given soil pressure [49]. On the other hand, the biomass allocated to a certain root component is also a function of the ramifications and/or their diameter, suggesting that studying biomass assigned to the different root components can help to identify which component affects the resistance to pull-out and anchorage more. Ennos and Fitter [51] showed that various anchorage strategies (plate-like and tap-like morphology) have an impact on biomass allocation patterns, therefore stressing the necessity of studying BGB allocation patterns to understand anchorage strategies.

Bila et al. [52] have demonstrated that silvicultural treatments positively influenced the health and growth of *A. johnsonii* and suggested that this species can be grown for commercial and urban forestry purposes. Knowledge of the extent and distribution of tree root systems is essential for managing trees in the constructed environment [53]. Conversely, the performance of urban trees depends upon the ability of their root systems to acquire resources and provide anchorage [53]. This emphasises the requirement to study BGB allocation patterns, though the anchorage of open grown trees and of those grown in the woods may be different.

Hence, as direct estimation of BGB is labour- and time-intensive, developing root component biomass models based on easily measurable variables is crucial for studying BGB allocation patterns and therefore anchoring functions of the different root components.

The present study was aimed at analysing the BGB allocation patterns, fitting and validating root component and root system models for *A. johnsonii*. A general model of the root system was also fitted using the best root component models using weighted non-linear seemingly unrelated regression (WNSUR) and critically compared against independent root system models. The excavation depth range that can capture more than 75% of the BGB was also estimated.

## Results

### Description of the data

**Figure 1:**
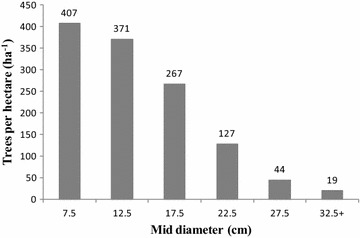
Diameter distribution histogram of phase-1 sampled *A. johnsonii* trees.

Diameter distributions of the phase-1 and phase-2 trees are given in the Figure 1 and Table 1, respectively, and show that the phase-2 sample trees (outside the brackets in the Table 1) are representative of those from phase-1. On the other hand, it is also noted that the testing sample trees (inside the brackets in the Table 1) are also representative of those from phase-2.

**Table 1:** Diameter distribution of the training sample trees (outside the brackets) and of the testing sample trees felled inside the study area (inside the brackets)

Diameter class (cm)	Manjacaze/Chibuto	Mabote	Funhalouro	Total
[05–10[	3 (1)	6 (2)	9 (2)	18 (5)
[10–15[	3 (1)	7 (2)	8 (2)	18 (5)
[15–20[	4 (1)	6 (0)	8 (2)	18 (3)
[20–25[	4 (1)	5 (1)	8 (1)	17 (3)
[25–30[	3 (0)	6 (2)	8 (2)	17 (4)
[30–35]+	3 (1)	1 (0)	1 (0)	5 (1)
Total	20 (5)	31(7)	42 (9)	93 (21)

**Figure 2:**
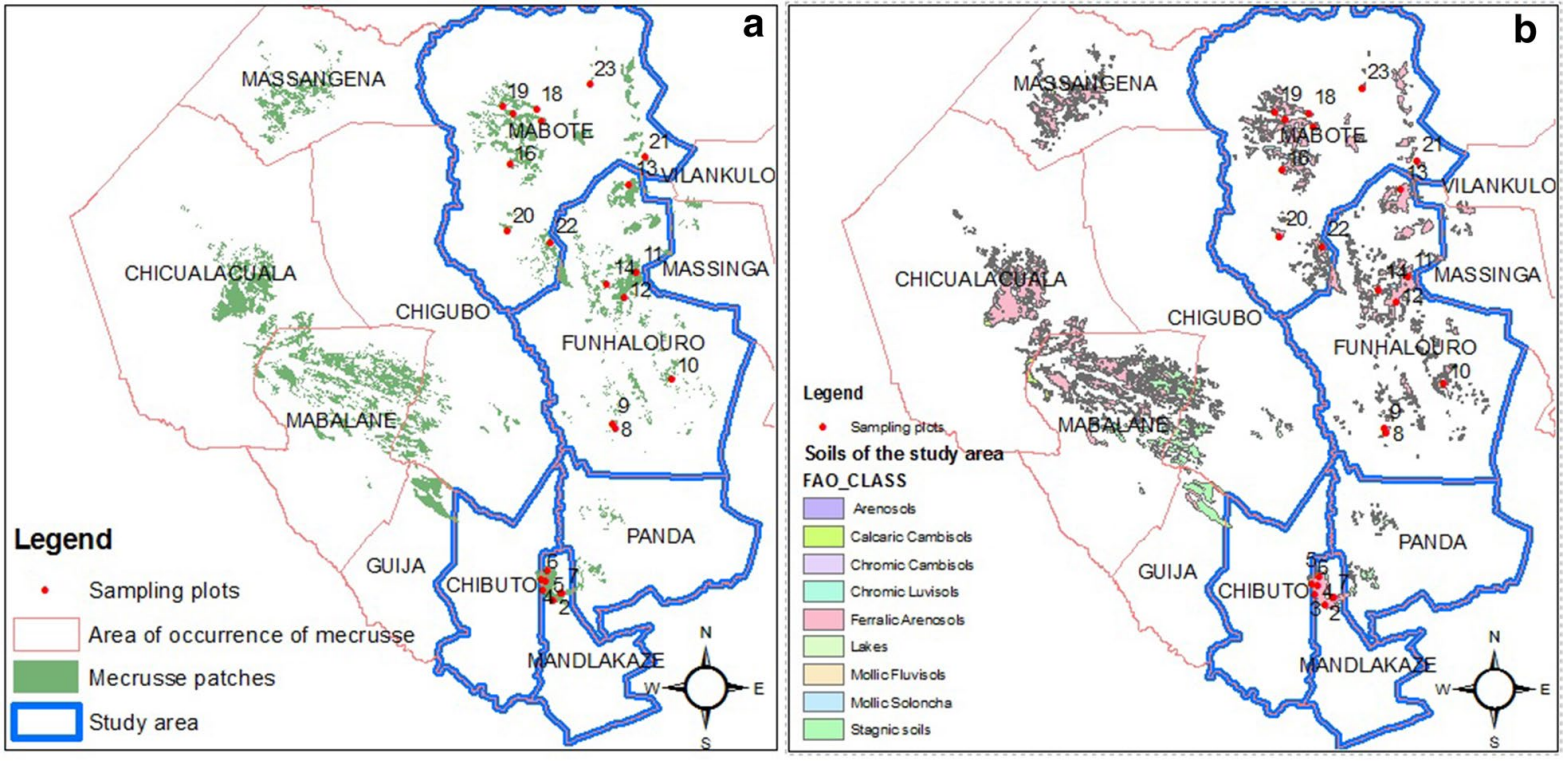
Area of occurrence of *A. johnsonii* in the districts of Gaza and Inhambane Provinces (**a**) and its soil types (**b**).

The testing sample collected outside the study area (in Chicualacuala district) was distributed according to diameter classes in the Table 1, as follows: 4, 3, 3, 3, 2 and 1, respectively. Note that, although the study area comprised 5 districts (Chibuto, Madlakaze, Panda, Funhalouro and Mabote), during the randomization (Figure 2), none of the plots fell in Chibuto and Panda districts, and those districts have almost a negligible share of mecrusse woodlands of the study area.

### BGB allocation patterns

On average, the percentage of the root system biomass attributed to taproot, root collar, and lateral roots biomasses was 48.36, 30.79 and 51.64%, respectively; and the percentage of the taproot attributed to root collar was 64.89% (Table 2). The percentage of the root system biomass found at 20% of the taproot depth, which is equivalent to 9.6–61.2 cm in depth from the ground level, was 81.20%.

**Table 2:** Belowground biomass allocation patterns

Statistic	Taproot (TR)		Root collar (RC)			Lateral roots (LR)		Root system (RS) (Kg)	% of the RS found at 20% of the TR depth
	Kg	% RS_TR_	Kg	% TR_RC_	% RS_RC_	Kg	% RS_LR_		
Minimum	0.00	0.00	0.00	2.86	0.00	0.00	0.00	1.93	40.43
Average	18.64	48.36	13.32	64.92	30.79	24.05	51.64	42.69	81.20
Maximum	63.60	100.00	54.90	89.61	55.86	100.82	100.00	149.38	100.00
SD	15.78	15.34	12.29	16.64	11.22	23.98	15.34	37.97	11.16
CV	84.65	31.72	92.26	25.64	36.44	99.69	29.71	88.94	13.53

The last column represents the percentage of the root system biomass found at 20% of the taproot depth, which is equivalent to 9.6 to 61.2 cm in depth from the ground level.

*SD* standard deviation, *CV* coefficient of variation (%), *% RS*_*TR*_ percentage of the root system biomass attributed to the taproot biomass, *%TR*_*RC*_ percentage of the taproot biomass attributed to the root collar biomass, *%RS*_*RC*_ percentage of the root system biomass attributed to root collar biomass, *%RS*_*LR*_ percentage of the root system biomass attributed to lateral roots biomass.

Table 3 shows that BGB allocation patterns vary with tree size (DBH, RCD, and TH), except the proportion of root system biomass allocated to the root collar (RC/RS), which is found to be independent on tree size by either Pearson´s correlation test or dcov test of independence.

**Table 3:** Dependence of BGB allocation patterns on tree size

No.	Pair of variables	Pearson’s correlation test		Distance covariance test of independence		
		r	p value	dcov	dcor	p value
1	LR/RS vs. DBH	0.4120	4.08E−05	0.3388	0.4856	0.0150
2	TR/RS vs. DBH	−4.3129	4.08E−05	0.3388	0.4856	0.0150
3	RC/RS vs. DBH	0.0662	0.5285^ns^	0.1118	0.1841	0.2200^ns^
4	RC/TR vs. DBH	0.5816	9.7E−10	0.4451	0.5851	0.0150
5	LR/RS vs. RCD	0.4078	5.0E−05	0.3357	0.4722	0.0150
6	TR/RS vs. RCD	−0.4078	5.0E−05	0.3357	0.4722	0.0150
7	RC/RS vs. RCD	0.0942	0.3689^ns^	0.1168	0.1888	0.1650^ns^
8	RC/TR vs. RCD	0.6051	1.3E−10	0.4653	0.6002	0.0150
9	LR/RS vs. TH	0.2662	0.0099	0.1423	0.4040	0.0150
10	TR/RS vs. TH	−0.2662	0.0099	0.1423	0.4040	0.0150
11	RC/RS vs. TH	0.1451	0.1654^ns^	0.0775	0.2528	0.0540^ns^
12	RC/TR vs. TH	0.5418	0.0000	0.2093	0.5446	0.0150

*r* Pearson’s correlation coefficient, *dcov* distance covariance, *dcor* distance correlation, *ns* not statistically significant at α = 0.05.

### Modelling

The laterals roots and root system biomass models showed that more than 99% of the BGB variation was explained by the predictor variables (Tables 4, 5). The root collar and taproot biomass models showed that more than 96 and 98% of the BGB variation, respectively, were explained by the predictor variables. The CVr varied from, approximately, 23 to 46%; the smallest and highest CVr values were verified for the root system and the root collar biomass models, respectively.

**Table 4:** Coefficients of regression (± standard error) for independently fitted models

Model form #	Weight function	b_0_ (±SE)	b_1_ (±SE)	b_2_ (±SE)
Root collar				
1	1/0.0002 × DBH^3.6194^	0.0129 (±0.0040)**	2.3350 (±0.1024)***	
2	1/0.0001 × DBH^3.6750^	0.0035 (±0.0030)^ns^	2.0979 (±0.1606)***	0.7807 (±0.4478)^ns^
3	1/0.0358 × exp(0.2527 × DBH)	0.0024 (±0.0010)*	1.0143 (± 0.0497)***	
4	1/0.0352 × exp(0.2219 × RCD)	0.0064 (±0.0024)**	2.4946 (± 0.1216)***	
Taproot				
1	1/0.0002 × DBH^3.7383^	0.0427 (±0.0103)***	2.0594 (±0.0840)***	
2	1/0.0002 × DBH^3.7521^	0.0092 (±0.0066)^ns^	1.7587 (±0.1447)***	0.9442 (±0.3989)*
3	1/0.0001 × DBH^3.8793^	0.0101 (±0.0029)***	0.8885 (±0.0353)***	
4	1/0.0002 × RCD^3.6082^	0.0269 (±0.0074)***	2.1454 (±0.0910)***	
Lateral roots				
1	1/0.000008 × DBH^4.8739^	0.0099 (±0.0021)***	2.6041 (±0.0748)***	
2	1/0.00007 × DBH^4.0862^	0.0045 (±0.0028)^ns^	2.5257 (±0.1213)***	0.3983 (±0.3265)^ns^
3	1/0.00006 × DBH^4.1635^	0.0012 (±0.0004)**	1.1585 (±0.0369)***	
4	1/0.0189 × exp(0.2826 × RCD)	0.0038 (±0.0011)***	2.8352 (±0.0975)***	
Root system				
1	1/0.00007 × DBH^4.3051^	0.0405 (±0.0064)***	2.3402 (±0.0545)***	
2	1/0.000008 × DBH^1.7645^	0.0185 (±0.0064)**	2.1990 (±0.0793)***	0.4699 (±0.1898)*
3	1/0.0003 × DBH^3.8639^	0.0070 (±0.0015)***	1.0226 (±0.0251)***	
4	1/0.00007 × RCD^4.2484^	0.0230 (±0.0042)***	2.4519 (±0.0608)***	

*SE* standard error, *ns* not statistically significant at α = 0.05.

* Significant at α = 0.05.

** Significant at α = 0.01.

*** Significant at α = 0.001.

**Table 5:** Goodness of fit statistics, and heteroskedasticity and normality tests of residuals for independently fitted models

Model form #	Adj.R^2^ (%)	CVr (%)	MR (%)	White,s test for heteroskedasticity		Lilliefors normality test	
				χ^2^	p value	D	p value
Root collar							
1	98.22	42.91	−0.6582^ns^	10.2673	0.5925	0.0917	0.0518
2	97.02	44.85	−0.9926^ns^	9.4548	0.6637	0.0761	0.2048
3	97.43	44.80	−1.3605^ns^	8.9433	0.7078	0.0783	0.1728
4	96.98	45.62	−1.4202^ns^	11.0543	0.5243	0.0800	0.1508
Taproot							
1	98.55	37.71	−0.0437^ns^	5.2695	0.9484	0.0420	0.9537
2	98.68	37.21	−0.1412^ns^	3.5465	0.9903	0.0516	0.7859
3	98.16	38.85	−0.2415^ns^	3.6553	0.9889	0.0413	0.9608
4	98.57	37.50	0.0035^ns^	8.6969	0.7286	0.0621	0.5066
Lateral roots							
1	99.40	35.8213	0.0589^ns^	7.9210	0.7913	0.0801	0.1504
2	99.44	33.8683	1.1136^ns^	6.0923	0.9114	0.0873	0.0771
3	99.45	31.5039	0.1433^ns^	7.1091	0.8503	0.1289	0.0006
4	99.26	38.5273	1.6894^ns^	6.3422	0.8979	0.0755	0.2146
Root system							
1	99.73	24.6597	0.0182^ns^	11.0001	0.5289	0.0562	0.6651
2	99.78	23.4060	0.1110^ns^	9.9195	0.6230	0.0498	0.8249
3	99.80	23.1551	1.3768^ns^	12.5144	0.4053	0.0470	0.8808
4	99.79	28.8790	0.1124^ns^	16.2835	0.1786	0.0480	0.8620

*D* Lilliefors statistic, *ns* not statistically significant at α = 0.05.

All the root component models presented statistically insignificant bias (MR) as tested by Student’s *t*-test and their residuals showed homoscedasticity (*p*-value >> 0.05) and normal distribution (*p*-value > 0.05) (except for the model form 3 of the lateral roots) (Table 5). The plots of the residuals (not shown) presented no particular trend; the cluster of points was contained in a horizontal band, with the residuals evenly distributed under and over the axis of abscissas, meaning that there were not model defects. All the models performed almost equally.

### Forcing additivity of the taproot and lateral roots biomasses into root system biomass

Because the different model forms performed almost equally (Table 5), the WNSUR with parameter restriction was applied to models using DBH and TH as predictors; and to those using either DBH or RCD only. This ensured that the additivity could be achieved either using two variables (DBH and TH) or using only one variable (DBH or RCD). The latter case was included because TH is difficult and time-consuming to measure in natural forests.

The relatively better taproot and lateral roots model forms were the model forms (5) and (6) (refer to “Methods”), respectively, as judged by Adj.R^2^ and CVr, as other statistics were equally insignificant. Therefore, the root system model form is a function (sum) of the predictors of the models (5) and (6). The structural system of equations (including the root system biomass model) obtained by combining the best taproot and lateral roots model forms under parameter restriction is given in Eq. (1). Using the same principle for the model forms with either DBH or RCD only as predictors, the structural systems of equations for WNSUR are given in Eqs. (2) and (3).

1 $$\begin{aligned} &\hat{Y}_{Taproot} = b_{10} DBH^{{b_{11} }} TH^{{b_{12} }} \hfill \\ &\hat{Y}_{Lateral - roots} = b_{20} \left( {DBH^{2} TH} \right)^{{b_{21} }} \hfill \\ &\hat{Y}_{Root - system} = b_{10} DBH^{{b_{11} }} TH^{{b_{12} }} + b_{20} \left( {DBH^{2} TH} \right)^{{b_{21} }} \hfill \\ \end{aligned}$$

2 $$\begin{aligned} &\hat{Y}_{Taproot} = b_{10} DBH^{{b_{11} }} \hfill \\ &\hat{Y}_{Lateral - roots} = b_{20} DBH^{{b_{21} }} \hfill \\ &\hat{Y}_{Root - system} = b_{10} DBH^{{b_{11} }} + b_{20} DBH^{{b_{21} }} \hfill \\ \end{aligned}$$

3 $$\begin{aligned} &\hat{Y}_{Taproot} = b_{10} RCD^{{b_{11} }} \hfill \\ &\hat{Y}_{Lateral - roots} = b_{20} RCD^{{b_{21} }} \hfill \\ &\hat{Y}_{Root - system} = b_{10} RCD^{{b_{11} }} + b_{20} RCD^{{b_{21} }} \hfill \\ \end{aligned}$$

Note that, in Eqs. (1–3), the coefficients of regression of each regressor in each root component model (taproot or lateral roots) are forced (constrained, restricted) to be equal to coefficients of the equivalent regressor in the root system model, allowing additivity.

For the WNSUR in Eqs. (1–3) the range of Adj.R^2^ is 83.30–93.46, 82.59–93.32, and 82.92–92.90%, respectively (Tables 6, 7). It was observed that by forcing additivity the Adj.R^2^ decreased, and the normality of the residuals was lost (*p*-value < 0.05). However, the bias (MR) kept insignificant and the residuals showed homoscedasticity (*p*-value >> 0.05). The models in Eqs. (1–3), fitted under WNSUR, performed almost equally.

**Table 6:** Coefficients of regression (± standard error) for simultaneously fitted models

Root component	Weight function	b_10_ (±SE)	b_11_ (±SE)	b_12_ (±SE)	b_20_ (±SE)	b_21_ (±SE)
Using DBH and TH as independent variables						
Taproot	1/0.0002 × DBH^3.7421^	0.0101 (±0.0072)^ns^	1.7737 (±0.1439)***	0.8910 (±0.3949)*		
Lateral roots	1/0.0002 × DBH^3.7421^				0.0011 (±0.0004)*	1.1703 (±0.385)***
Root system	1/0.0002 × DBH^3.7421^	Taproot model + lateral roots model				
Using only DBH as independent variable						
Taproot	1/0.0002 × DBH^3.6194^	0.0430 (±0.0106)***	2.0567 (±0.0849)***			
Lateral roots	1/0.0002 × DBH^3.6194^				0.0090 (±0.0024)***	2.6368 (±0.0252)***
Root system	1/0.0002 × DBH^3.6194^	Taproot model + lateral roots model				
Using only RCD as independent variable						
Taproot	1/0.0002 × RCD^3.6082^	0.0269 (±0.0074)***	2.1454 (±0.0910)***			
Lateral roots	1/0.0002 × RCD^3.6082^				0.0048 (±0.0014)**	2.7624 (±0.0947)***
Root system	1/0.0002 × RCD^3.6082^	Taproot model + lateral roots model				

*SE* standard error, *ns* not statistically significant at α = 0.05, * Significant at α = 0.05, ** Significant at α = 0.01, *** Significant at α = 0.001.

**Table 7:** Goodness of fit statistics, and heteroskedasticity and normality tests of residuals for simultaneously fitted models

Root component	Adj.R^2^ (%)	CVr (%)	MR (%)	White´s test for heteroskedasticity		Lilliefors normality test		$\hat{\sigma }_{ii}$	$\hat{\sigma }_{WNSUR}^{2}$
				χ^2^	p-value	D	p-value		
Using DBH and TH as independent variables									
Taproot	83.30	39.07	−0.2625^ns^	13.7327	0.3181	0.1348	0.0003	3.2284	1.9462
Lateral roots	90.26	31.34	4.9056^ns^	19.7679	0.0716	0.1299	0.0005	2.8909	
Root system	93.46	22.95	1.4469^ns^	13.1392	0.3590	0.1450	5E−05	5.8425	
Using only DBH as independent variable									
Taproot	82.59	37.91	−0.0590^ns^	15.1331	0.2342	0.1185	0.0026	4.9418	1.9570
Lateral roots	90.53	35.78	0.6559^ns^	18.2943	0.1070	0.1537	1E−05	4.2161	
Root system	93.32	24.69	0.1103^ns^	11.7599	0.4652	0.1403	1E−04	8.8881	
Using only RCD as independent variable									
Taproot	82.92	41.27	−0.0065^ns^	18.3541	0.1054	0.1289	0.0006	3.5120	1.9570
Lateral roots	90.34	38.07	0.4631^ns^	17.2169	0.1416	0.1836	3E−08	3.2507	
Root system	92.80	29.11	0.0843^ns^	14.7963	0.2528	0.1696	6E−07	7.1575	

*ns* not statistically significant at α = 0.05, *D* Lilliefors statistic, $\hat{\sigma }_{ii}$ the (i, i) element of the covariance matrix of the residuals $\hat{\varSigma }$ (error covariance matrix), it is the covariance error of the *ith* system equation, $\hat{\sigma }_{WNSUR}^{2}$ WNSUR system variance.

The *t*-test results for the restrictions imposed on WNSUR (Table 8) were insignificant (p-value ≈1), indicating that the data were consistent with the restriction and that the models fit as well with the restriction imposed.

**Table 8:** *t* test for the restrictions imposed for WNSUR

Restriction	Parameter estimate	Standard error	t value	Pr > |t|
Using DBH and TH as independent variables				
Rest1	1.1859	30789.70	0.00	1.0000
Rest2	0.0102	287.40	0.00	1.0000
Rest3	1.7386	2664.90	0.00	0.9995
Rest4	0.0456	77.54	0.00	0.9995
Rest5	0.0451	68.64	0.00	0.9995
Using only DBH as independent variable				
Rest1	5.3731	3710.80	0.00	0.9989
Rest2	0.1517	102.70	0.00	0.9988
Rest3	0.1960	612.20	0.00	0.9987
Rest4	0.0215	76.40	0.00	0.9988
Using only RCD as independent variable				
Rest1	16.9897	6901.20	0.00	0.9981
Rest2	0.2539	104.50	0.00	0.9981
Rest3	0.1317	986.70	0.00	0.9999
Rest4	0.0112	80.31	0.00	0.9999

Rest1 to Rest5 are the restrictions imposed to each of the regression coefficients, as stated in Eqs. (1–3).

### Validation

The aggregate difference (AD) for independent models (Table 9) varied from −6.06 to 0.21% in the study area and from 0.98 to 6.0 outside the study area. For the whole testing sample (including both the inside and outside samples) the AD varied from −2.9 to 5.1%. For simultaneous models the range of AD was from −5.4 to 0.68% and from 3.42 to 5.90%, inside and outside the study area, respectively (Table 10). For the whole testing sample the AD ranged from −2.87 to 5.1 %. The Wilcoxon signed rank test revealed that for both independent and simultaneous models (Tables 9, 10) the observed BGB did not differ statistically from the predicted BGB values (p-value >0.25); hence, the models can be used reliably inside and outside the study area.

**Table 9:** Validation of independently fitted models

Model form #	Inside the study area (n = 21)			Outside the study area (n = 16)			Total (n = 37)		
	AD (%)	*V*	p-value	AD (%)	*V*	p-value	AD (%)	*V*	p-value
Root collar									
1	−3.8062	144.0	0.3377	0.9789	61.0	0.7436	−1.4127	399.0	0.6880
2	−2.9985	146.0	0.3038	2.6842	61.0	0.7436	−1.6542	418.0	0.4998
3	−2.2222	141.0	0.3926	3.6755	60.0	0.7057	−1.1550	409.0	0.5856
4	−6.0617	120.0	0.8917	2.8364	64.0	0.8603	−2.3628	380.0	0.8974
Taproot									
1	−2.3818	127.0	0.7079	4.1225	63.0	0.8209	−0.4035	392.0	0.7634
2	−1.7091	121.0	0.8649	5.7234	63.0	0.8209	−0.8994	378.0	0.9201
3	−1.6672	121.0	0.8649	5.7082	63.0	0.8209	−0.7959	378.0	0.9201
4	−5.3688	123.0	0.8117	3.5819	64.0	0.8603	−2.8804	397.0	0.7093
Lateral roots									
1	−1.1701	119.0	0.9187	2.7810	50.0	0.3755	4.3074	317.0	0.4465
2	0.1249	117.0	0.9729	4.4842	48.0	0.3225	5.0567	310.0	0.3885
3	0.2054	118.0	0.9457	5.4708	47.0	0.2979	4.2389	304.0	0.3425
4	−3.8743	139.0	0.4319	5.9664	62.0	0.7820	3.7639	375.0	0.9543
Root system									
1	−1.7047	131.0	0.6091	3.4307	52.0	0.4332	2.2047	346.0	0.7308
2	−1.2378	123.0	0.8117	4.4345	52.0	0.4332	2.0331	327.0	0.5371
3	−0.8691	113.0	0.9457	5.3854	48.0	0.3225	1.7691	318.0	0.4551
4	−5.4699	129.0	0.6578	3.6655	61.0	0.7436	−0.3734	366.0	0.9543

*AD* aggregate difference, *V* Wilcoxon statistic.

**Table 10:** Validation of simultaneously fitted models

Root component	Inside the study area (n = 21)			Outside the study area (n = 16)			Total (n = 37)		
	AD (%)	*V*	p-value	AD (%)	*V*	p-value	AD (%)	*V*	p-value
Using DBH and TH as independent variables									
Taproot	−1.7243	121.0	0.8649	5.6595	63.0	0.8209	−0.8602	378.0	0.9201
Lateral roots	0.6760	120.0	0.8917	5.8973	46.0	0.2744	4.7203	303.0	0.3352
Root system	−0.3711	117.0	0.9729	5.7933	49.0	0.3484	2.2976	319.0	0.4639
Using only DBH as independent variable									
Taproot	−2.4598	127.0	0.7079	4.0529	63.0	0.4037	−0.4861	393.0	0.7524
Lateral roots	−0.4410	119.0	0.9187	3.4277	51.0	0.8209	5.0618	319.0	0.4639
Root system	−1.3237	134.0	0.5392	3.7025	57.0	0.5966	2.6531	349.0	0.7634
Using only RCD as independent variable									
Taproot	−5.3576	123.0	0.8117	3.5923	64.0	0.8603	−2.8693	397.0	0.7093
Lateral roots	−5.3553	134.0	0.5392	4.3072	63.0	0.8209	2.0879	370.0	1.0000
Root system	−5.3563	131.0	0.6091	3.9955	61.0	0.7436	−0.0734	369.0	0.9886

*AD* aggregate difference, *V* Wilcoxon statistic.

## Discussion

### BGB allocation patterns

Considering the fact that 90% of the lateral roots of *A. johnsonii* trees are located in the first node, which is located close to the ground level [16, 17, 38, 50] it can be inferred that the 81.20% of the root system (found up to 61.2 cm in depth) is composed by root collar and lateral roots and therefore, the remaining portion of the taproot constitutes less than 20% of the root system biomass. This can be verified by summing the average taproot and lateral roots biomasses, which is equal to 82.43%, very close to the percentage of the root system biomass found at 20% of the taproot depth (81.20%). The difference of those percentages (1.13%) represents the lateral roots found at depths above 20% of the taproot depth (above the depth range of 9.6–61.2 cm from ground level).

Depths of excavation of the roots are not standardized [18, 34], but the depth selected in a given study is assumed to capture a large portion of the roots [18]. For example, Green et al. [19], Kuyah et al. [23], Sanquetta et al. [30], and Paul et al. [31], used excavation depths of 50–120, 40–200, 50, and <200 cm, respectively. Schenk and Jackson [54] found that globally, 95% of all roots are within the upper 200 cm of the soil profile. In this study, the excavation range (9.6–61.2 cm) that captured 81.20% of root biomass fall in the excavation range by those authors.

However, BGB estimates by Green et al. [19], Kuyah et al. [23], Sanquetta et al. [30], and Paul et al. [31] might have been underestimated, as according to Mokany et al. [20], sampling to insufficient soil depth to capture the majority of the roots, not sampling the root collar, not sampling fine roots, and sampling with inadequate replication are some methodological pitfalls associated with sampling root biomass, and can lead to underestimation. In this study, the root system was excavated to total depth and removed, including the root components generally ignored as stated by Mokany et al. [20].

The proportion of BGB allocated to the root collar (average = 30.79%, CV = 36.44%) is lower than that reported by Mokany et al. [20] (average = 41%, CV = 3.10%). Inclusion of different terrestrial biomes by Mokany et al. [20] may justify this discrepancy.

It is noted from Table 3 that as the tree increased in DBH, RCD, and TH, the proportion of BGB allocated to taproots (TR/RS) decreased and that allocated to lateral roots (LR/RS) increased. This is presumably because as the trees grow the larger is the need for its anchorage in the soil; and that the anchorage function in larger *A. johnsonii* trees is much attributed to the lateral roots as they hold a larger amount of soil than the taproots. In fact, there are trees without taproots but hardly a tree can sustain itself in the soil without lateral roots, especially the larger ones. As maintained by Crook and Ennos [55] only relatively small tree species can rely solely on the taproot for anchorage and that this is the reason that most large trees develop thick lateral roots. Moreover, Bailey et al. [56] have verified that lateral roots play an important role in anchorage.

It has also been noted by Dupuy et al. [39] that heart like root systems (those root systems that possess large lateral roots originating from the centre of the bole) generally had the most efficient anchorage. The heart like root system is determined by lateral roots, implying that the lateral roots influence greatly the anchorage efficiency. *A. johnsonii* trees do not have larger lateral roots than the taproot; however, they have many lateral roots (up to 11 lateral roots per node) which make them to contain a larger proportion of biomass than the taproot (Table 2). In fact, Dupuy et al. [40] maintained that the number of lateral roots is determinant for anchorage, as they determine the root’s ability to bear a large amount of soil and the area of soil mobilized during pull-out.

Trees are also known to respond to wind stress by increasing the number of lateral roots, which provide the most resistance to overturning [57]. This emphasizes the importance of lateral roots for tree anchorage, hence larger allocation of BGB biomass to them (Table 2), especially as trees grow (Table 3).

The decreasing BGB allocation to taproot with tree size might be related to decreasing anchorage function of the taproot with tree size. Khuder et al. [58] argued that “taproots may play an important role in anchoring young trees, but in adult trees, their growth is often impeded by the presence of a hard pan layer in the soil and the taproot becomes a minor component of tree anchorage”.

### Modelling, additivity, and validation

The homoscedasticity observed for the independent and simultaneous models implies that the derived weight functions were efficient in addressing the heteroskedasticity.

Because all the fitted independent models performed almost equally, any model can be used accurately to estimate BGB and carbon stocks in *A. johnsonii* stands (mecrusse woodlands), which, along with mopane and miombo are the most important woodlands in Gaza and Inhambane Provinces. However, as including tree height improved the fit statistics of the models negligibly, and sometimes worsened them, the models using either DBH or RCD only should be preferred, as tree height is difficult to measure in natural forests. The model using the RCD only should be preferred when the tree is found cut down, as besides RCD being relatively difficult to measure compared to DBH, it is sometimes influenced by root buttress. The same holds true for simultaneous models, as they also performed equally.

The mean biomass per tree (MB) obtained using the WNSUR root system model based on DBH and TH [Eq. (1)], based on DBH [Eq. (2)], and based on RCD [Eq. (3)] was only 0.40, 0.31 and 0.23% larger than the MB obtained using the independent root system model based on DBH and TH (model form 6, found to be the best), based on DBH, and based on RCD, respectively. The differences between the MB of the root components (taproot and lateral roots) obtained using simultaneous and independent models were also negligible (>0.50%). These results are in line with those found by Repola [7].

As the differences between MB obtained using simultaneous and independent models were negligible, the independent models should be preferred to avoid unnecessary complexity of the models, and also because the residuals for simultaneously fitted models were not normally distributed.

This study is distinguished from other studies that include BGB (e.g. [6, 15, 19, 23, 29, 31, 33]) for five reasons: (1) in this study a very large sample size and geographical range was covered (93 trees of a single species harvested in 5 districts); (2) the root system was excavated to total depth and removed (including fine roots); (3) the root system was divided into three root components (taproot, root collar, and lateral roots) allowing, therefore, fitting the models for each root component and analysing BGB allocation patterns; (4) the error variance was modelled to derive the weight functions and address heteroskedasticity, which led to increases in Adj.R^2^ and general improvement of the models´ performance and; (5) the models were validated inside and outside the study area, therefore, the predictive capacity of the models was checked.

Mugasha et al. [6] used a large number of sample trees (80) distributed over 60 tree species, making up an average of 1.34 trees per species, which might not have been representative. Moreover, when modelling tree biomass, the use of species-specific equations are preferred because trees of different species may differ greatly in architecture and wood density [59], and architecture can influence biomass allocation and allometry [41, 42].

Mugasha et al. [6] and Kuyah et al. [23] did not fully excavated the root system, relied on sampling procedures, which might have led to less accurate estimates when compared to cases where all the root system is removed. Green et al. [19], Kuyah et al. [23], Ruiz-Peinado et al. [29], Paul et al. [31], and Ryan et al. [33] used predefined excavation depths and/or did not include fines roots; which leads to underestimation of the BGB [20].

Mugasha et al. [6], Litton et al. [15], Green et al. [19], Ruiz-Peinado et al. [29], and Ryan et al. [33] did not validate their models, therefore, the predictive capacity of the models was not checked. Paul et al. [31] checked the predictive capacity of the models only in the study area. Kuyah et al. [23] used a very small testing sample (6 trees) to validate the models which obviously did not cover all variation ranges.

The fit statistics for BGB model by Magalhães and Seifert [38] using the same model form as the one in Eq. (3) (Adj.R^2^ = 94.94%; CVr = 21.79%) were different from those obtained here (Adj.R^2^ = 99.80%; CVr = 23.16%). These differences might be because Magalhães and Seifert [38] considered the stump as part of the root system and because the weight functions were derived interactively; the weight functions were, therefore, just approximations.

Independent BGB (root system) models of this study (Adj.R^2^ range 99.73–99.80%; bias range 0.02–1.38%) performed better than those by Kuyah et al. [23] (R^2^ range 91.90–96.10%; bias range −49.60 to 35.40%) and Mugasha et al. [6] (R^2^ range 89.00–94.00%; bias range 0.12–5.98%). Models of this study also showed superiority to the BGB models by Paul et al. [31] (R^2^ range 64.00–95.00%) and Ryan et al. [33] (R^2^ = 94.00%).

Husch et al. [5] suggested that the aggregate difference should not exceed ${{2 \times CV_{r} } \mathord{\left/ {\vphantom {{2 \times CV_{r} } {\sqrt n }}} \right. \kern-0pt} {\sqrt n }}$, where *n* is the number of trees used in the test. In this study the lowest value of ${{2 \times CV_{r} } \mathord{\left/ {\vphantom {{2 \times CV_{r} } {\sqrt n }}} \right. \kern-0pt} {\sqrt n }}$ was 11.96%, which was almost twice as large as the largest aggregate difference; therefore, the models can be applied inside and outside the study area without requiring any corrections, according to this criterion.

The dataset of this study comprised a training and testing sample of 93 and 37 trees, respectively, with DBHs varying from 5 to 32 cm and from 5.5 to 32 cm, respectively. *A. johnsonii* trees hardly can exceed 35 cm in DBH. Magalhães and Soto [60] (unpublished data) found only 13 *A. johnsonii* trees per ha with DBH ≥ 30 cm, corresponding to only 5% of the total trees per ha. Here, the number of trees with DBH ≥ 32.5 cm were only 19 per ha, equivalent to 1.54% of the total trees per ha. This implies that no serious bias will be added when extrapolating the models outside the DBH range used to fit the models since very few trees are found outside that DBH range.

*A. johnsonii* stands occur mainly in Ferralic Arenosols and Stagnic soils (Figure 2b) [61], which cover 410,144 ha (74%) and 108,960 ha (20%), respectively, of the total area of occurrence of *A. johnsonii* stands (Figure 2a, b) in Gaza and Inhambane Provinces; the remaining 6% are covered by other soil types [61]. Of the 23 sampled plots, 20 were located in Ferralic Arenosols and 3 (plots 8, 9 and 22 in Figure 2b) were located in Stagnic soils. The 20 plots from Ferralic Arenosols accounted with 81 felled trees of the training sample, the remaining 12 trees were from Stagnic soils. The testing sample trees from outside the study area (16 trees from Chicuacala district) were all from Ferralic Arenosols (Figure 2b). The climate in the districts where *A. johnsonii* occurs is dry and humid tropical [2, 3, 61–71], however, *A. johnsonii* occurs only in dry tropical climates [61]. Humid tropical climate occurs only along the coast, where *A. johnsonii* stands do not occur [2, 3, 61–71]. This implies that the fitted models can also be safely applicable and valid over a vast range of soils and regions where *A. johnsonii* occurs and outside the study area.

Therefore, besides the fact that the models were validated inside and outside the study area, the study area covered almost the entire range of soil and climate variations where *A. johnsonii* occurs (despite the apparent lack of large variations), and a wide range of DBHs and THs, therefore, the models can be applied in all *A. johnsonii* stands in Mozambique.

## Conclusions

In this study, it was found that more than half and almost one-third of BGB is allocated to the lateral roots and to the root collar, respectively. More than 80% of the BGB was found at a depth range of 9.6–61.2 cm from the ground level. As the tree size increased, the proportion of BGB allocated to taproots decreased and that allocated to lateral roots increased. Consequently, it was hypothesised that BGB allocation patterns is a response of the anchorage functions of the tap and lateral roots and therefore, root component biomass models can be used as a methodology to predict anchoring functions of the different root components.

Because all fitted independent models performed almost equally, the models using either DBH or RCD exclusively are preferred as tree height is difficult to measure in natural forests. The model using RCD only as a predictor variable should be further preferred when the tree is found cut down, as sometimes the RCD is affected by root buttress. As a result of the differences between the mean biomasses obtained using independent and simultaneous models being negligible, the independent models should be preferred to avoid unnecessary complexity in the models.

The fitted independent models were based on a very large sample size (93 trees) and a wide geographical range (5 districts) and exhibited that, on average, 98% of the variation in BGB is explained by the predictor variables and were validated inside and outside the study area. Therefore, the models presented here could be applied to all *A. johnsonii* stands in Mozambique.

## Methods

### Study area

The study was conducted in Mozambique. The study area comprised 5 districts (Mabote, Funhalouro, Panda, Mandlakaze, and Chibuto) of 2 provinces (Inhambane and Gaza) with an extension of 4,502,828 ha [61], of which 226,013 ha (5%) were *A. johnsonii* stands (Mecrusse). Mecrusse is a forest type where the main tree species, many times the only one, in the upper canopy is *A. johnsonii*. Detailed description of the species, forest type and study area can be found in Magalhães and Seifert [16, 17, 38, 50].

### Data acquisition

The data were collected in 2012 and 2014. In 2012, a two-phase sampling design was used to determine BGB. In the first phase, diameter at breast height (DBH), root collar diameter (RCD) and total tree height (TH) of 3574 trees were measured in 23 randomly located circular plots (20-m radius). Only trees with DBH ≥ 5 cm were considered. In the second phase, 93 trees (DBH range 5–32 cm; TH range 5.69–16 m), 2 to 6 per plot, were randomly selected from those analysed during the first phase for destructive measurement of BGB along with the variables from the first phase. In 2014, additional 37 trees (DBH range 5.5–32 cm; TH range 7.3–15.74 m) were felled outside sampling plots, 21 (DBH range 6.0–31 cm; TH range 9.37–15.74 m) inside and 16 (DBH range 5.5–32 cm; TH range 7.3–15.05 m) outside the study area (in Chicualacula district, Figure 2). The 93 trees collected in 2012 were used to fit BGB models (training sample) and those collected in 2014 (37 trees) were used to validate the models (testing sample).

Trees (both from 2012 and 2014) were cut down at 20 cm from the ground level. Thereafter, the root system was excavated and sampled as follows. First, the root system was partially excavated to the first node, using hoes, shovels, and picks; to expose the primary lateral roots. The primary lateral roots were numbered and separated from the taproot with a chainsaw and removed from the soil, one by one. This procedure was repeated in the subsequent nodes until all primary roots were removed from the taproot and the soil. Finally, the taproot was excavated and removed.

The removal of the root system to the total depth was relatively easy because 90% of the lateral roots of *A. johnsonii* are located in the first node, which is located close to ground level; the lateral roots grow horizontally to the ground level, do not grow downwards; and because the taproots had, at most, only 4 nodes and at least 1 node (at ground level). The root system was removed completely, so the depth of excavation depended on the depth of the taproot. For images illustrating the excavation process, refer to Magalhães and Seifert [16, 17, 38].

**Figure 3:**
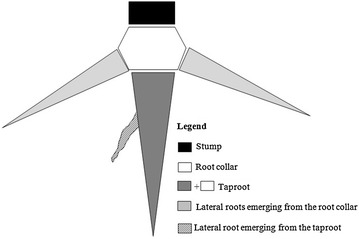
Scheme of the root system and its components.

The root system was divided into following root components: lateral roots (fine and coarse), root collar, and taproot. These root components are not additive, as the taproot includes also the root collar (Figure 3); therefore, the root system is the sum of lateral roots and taproot. The remaining portion of the taproot, obtained after removing the root collar, was not considered as an independent component because it would be an artificial component, as the taproot includes the root collar as well; moreover, there is no name for such a portion (Figure 3). Lateral roots with diameters at the insertion point on the taproot <5 cm were considered as fine roots and those with diameters ≥5 cm were considered as coarse roots.

Most of the studies on BGB (e.g. [15, 19, 23, 31, 72–75]) considered fine roots those with a diameter at insertion point on the taproot <2 mm and did not include them in the root samples. In this study, fine roots were defined as those with a diameter at insertion point on the taproot <5 cm and were included in the root samples. Therefore, although the definition of fine roots in this study is distinguished from that of most studies, the definition of fine roots by these authors is included in this study, and all dimensions of roots were considered here. Therefore, the definition of fine and coarse roots did not affect the estimates, as both definition categories were considered.

Fresh weight was obtained for the taproot, root collar, each coarse lateral root and for all fine lateral roots. A sample was taken from each root component, fresh weighed, marked, packed in a bag, and taken to the laboratory for oven drying. For the taproot, the samples were two discs, one taken on the top of the root collar and another from the middle of the taproot. For the coarse lateral roots, two discs were also taken, one from the insertion point on the taproot and another from the middle of it. For fine roots the sample was 5–10% of the fresh weight of all fine lateral roots. Oven drying of all samples was done at 105°C to constant weight, hereafter, referred to as dry weight.

Dry weights of the taproot, root collar, and lateral roots were determined by multiplying the ratio of oven-dry- to fresh-weight of each sample by the total fresh weight of the relevant component. The dry weight of the root system was obtained by summing the dry weights of taproot and lateral roots (fine and coarse ones). The disc taken on the top of the root collar was used to estimate its dry weight and the one taken in the middle of the taproot was used to estimate the dry weight of the remaining portion of the taproot.

### Data analysis

Possible variations of BGB allocation patterns with tree size (DBH, RCD, and TH) were studied by investigating the dependence of the ratios of lateral roots to root system (LR/RS), taproot to root system (TR/RS), root collar to root system (RC/RS), and root collar to taproot (RC/TR) biomasses on tree size, using Pearson’s correlation coefficient test of significance and distance covariance (dcov) test of independence. The first test was performed using the cor.test function of R software [76] and the second using the dcov.test function of energy package [77] in R software [76].

The Pearson’s correlation coefficient measures only linear dependencies. Although, the dcov test of independence measures all types of dependences (linear, non-linear and non-monotone) between random vectors X and Y in arbitrary dimension [78], the Pearson’s correlation coefficient was also considered, because unlike dcov test, it shows the direction of variation between two variables.

Biomass models were fitted using weighted non-linear regression. Non-linear models were preferred over linear ones because biomass is a non-linear function of stem diameter and height [32, 79–82]. Weighted least squares (WLS) were preferred over ordinary least squares (OLS) to address the heteroskedasticity as, quite often, the error variance is functionally related to the independent variables in regression [83], that is, the variability of the biomass increases with tree independent variables [84]. The weight functions were obtained by modelling the error structure as described by Parresol [83, 85]. For that, the squares of the OLS residuals were fitted against the different combination of the independent variables. Thus, it was assumed that the squares of the OLS residuals are representative of the error variance.

The tested model forms for all root components are given below.

4 $$\hat{Y} = b_{0} \times DBH^{{b_{1} }}$$

5 $$\hat{Y} = b_{0} \times DBH^{{b_{1} }} \times TH^{{b_{2} }}$$

6 $$\hat{Y} = b_{0} \times \left( {DBH^{2} \times TH} \right)^{{b_{1} }}$$

7 $$\hat{Y} = b_{0} \times RCD^{{b_{1} }}$$

where *Ŷ* is expressed in Kg, DBH and TH are expressed in cm and m, respectively.

A model form using the RCD only as a predictor variable was also considered to allow the estimate of BGB when trees are found already cut down and only stump dimensions are available. Estimation of BGB of exploited trees is very important, as in that case, BGB can be used to estimate the carbon that will be transferred to the soil and/or the nutrients that will be reclaimed by the site.

However, the sum of the biomass predictions for the root components will not equal the biomass prediction for the root system, and a desired and logical feature of the root component regression equations is that the sum of biomass predictions of the root components equals the prediction for the root system. To cope with that, a new root system biomass model form was obtained as a function of the predictor variables of the best taproot and lateral roots biomass model forms. Then, the new root system model form and the best taproot and lateral roots biomass model forms were fitted again, simultaneously, using weighted non-linear seemingly unrelated regression (WNSUR) with parameter restriction, to achieve additivity.

Root component models were fitted with the statistical software R [76] and the function nls using the Gauss–Newton algorithm. The simultaneous models (WNSUR models) were fitted using PROC MODEL statement of SAS software [86], using the ITSUR option. Restrictions on the regression coefficients were imposed by using RESTRICT statement. The start values of the parameters in nls function of the R software and in PROC MODEL statement of the SAS were obtained by fitting the logarithmized models of each component in Microsoft Excel.

The following criteria were used to evaluate the models (independent and simultaneous ones): adjusted coefficient of determination (Adj.R^2^), mean residual (MR), standard deviation of residuals (S_y.x_), test for heteroskedasticity of residuals, test for normality of residuals, and graphical analysis of residuals. The mean residual and the standard deviation of residuals were expressed as relative values, hereafter referred to as percent mean residual [MR (%)] and coefficient of variation of residuals [CV_r_ (%)], respectively, which are more revealing. MR measures the average model bias, describing the directional magnitude, the size of expected under and overestimates. The ideal value is zero. CV_r_ measures the dispersion between the observed and the estimated values of the model. It indicates the error that the model is subject to when is used for predicting the dependent variable. The ideal value is zero.

The heteroskedasticity of residuals was evaluated using the White’s test for heteroskedasticity, with the aid of the package het.test [87] of the R statistical software [76], under the null hypothesis of homoscedasticity. For that, the residuals were used as dependent variables and the predicted root component biomass as independent variable. The normality of residuals was evaluated using the Lilliefors normality test under the null hypothesis of normality, using the lillie.test function of R statistical software [76].

The models were then validated inside and outside the study area using aggregate difference in percentage (AD) [5, 88] and by comparing the observed and predicted BGB using Wilcoxon signed rank test in R [76] as recommended by Philip [89], under the null hypothesis of no difference between the observed and predicted BGB values. Aggregate difference is the prediction error of the models using an independent sample of trees (e.g. testing sample; trees not included in the sample used to fit the models).

All the statistical analyses were performed at α = 0.05.

## Abbreviations

AGB: aboveground biomass
BGB: belowground biomass
DBH: diameter at breast height
RCD: root collar diameter
TH: total tree height
WNSUR: weighted non-linear seemingly unrelated regression
AD: aggregate difference
MR: mean residual
CVr: coefficient of variation of residuals
Adj.R^2^: adjusted coefficient of determination
SE: standard error
dcor: distance correlation
dcov: distance covariance
WLS: weighted least squares
OLS: ordinary least squares
MB: mean biomass per tree
